# Revisiting Gauge-Independent Kinetic Energy Densities in Meta-GGAs and Local Hybrid Calculations of Magnetizabilities

**DOI:** 10.1021/acs.jpca.3c06244

**Published:** 2023-12-15

**Authors:** Caspar J. Schattenberg, Artur Wodyński, Hugo Åström, Dage Sundholm, Martin Kaupp, Susi Lehtola

**Affiliations:** † Institut für Chemie, Theoretische Chemie/Quantenchemie, Technische Universität Berlin, Sekr. C7, Straße des 17. Juni 135, D-10623 Berlin, Germany; ‡ Department of Chemistry, Faculty of Science, University of Helsinki, P.O. Box 55 (A.I. Virtanens plats 1), University of Helsinki FI-00014, Finland; § Molecular Sciences Software Institute, Blacksburg, Virginia 24061, United States

## Abstract

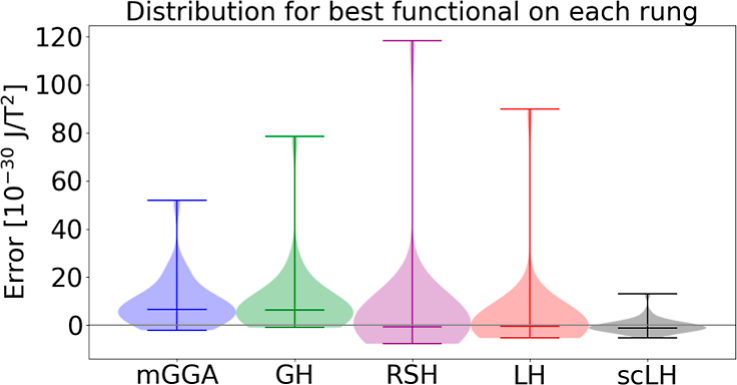

In a recent study [J. Chem. Theory Comput. 2021, 17, 1457–1468], some of us examined the accuracy of magnetizabilities calculated with density functionals representing the local density approximation (LDA), generalized gradient approximation (GGA), meta-GGA (mGGA), as well as global hybrid (GH) and range-separated (RS) hybrid functionals by assessment against accurate reference values obtained with coupled-cluster theory with singles, doubles, and perturbative triples [CCSD(T)]. Our study was later extended to local hybrid (LH) functionals by Holzer et al. [J. Chem. Theory Comput. 2021, 17, 2928–2947]; in this work, we examine a larger selection of LH functionals, also including range-separated LH (RSLH) functionals and strong-correlation LH (scLH) functionals. Holzer et al. also studied the importance of the physically correct handling of the magnetic gauge dependence of the kinetic energy density (τ) in mGGA calculations by comparing the Maximoff–Scuseria formulation of τ used in our aforementioned study to the more physical current-density extension derived by Dobson. In this work, we also revisit this comparison with a larger selection of mGGA functionals. We find that the newly tested LH, RSLH, and scLH functionals outperform all of the functionals considered in the previous studies. The various LH functionals afford the seven lowest mean absolute errors while also showing remarkably small standard deviations and mean errors. Most strikingly, the best two functionals are scLHs that also perform remarkably well in cases with significant multiconfigurational character, such as the ozone molecule, which is traditionally excluded from statistical error evaluations due to its large errors with common density functionals.

## 1 Introduction

Molecular magnetic properties such as nuclear magnetic resonance (NMR) shielding constants, NMR spin–spin coupling constants, and magnetizabilities are useful probes of molecular and electronic structure, and they can be studied to pinpoint the location of atoms in a molecule, for instance. However, interpretation of the measured spectra often requires computational modeling. Quantum chemical modeling of magnetic properties is often pursued using Kohn–Sham (KS) density functional theory^1,2^ (DFT).^3−10^ However, the accuracy of DFT for some of these properties has not been thoroughly established in the literature. Some of us recently employed the benchmark set of ref (7) to assess the accuracy of density functionals^11,12^ with an emphasis on newer functionals from rungs 2–4 of the usual Jacob’s ladder hierarchy;^13^ this benchmark set consists of magnetizabilities for 28 small main-group molecules computed with coupled-cluster theory with singles, doubles, and perturbative triples [CCSD(T)], which is a highly accurate wave function theory (WFT).

It was found in ref (11) that the BHandHLYP^14^ global hybrid (GH) functional and some range-separated hybrid (RSH) functionals provided the closest agreement with the CCSD(T) reference data, with the smallest mean absolute deviations (MADs) being slightly above 3 × 10^–30^ J/T^2^, while Hartree–Fock gave 7.22 × 10^–30^ J/T^2^ and was ranked 29th best out of 52 methods evaluated.^11,12^ Some functionals, in particular, the highly parameterized Minnesota functionals, were found to reach MADs with large errors above 10 × 10^–30^ J/T^2^.

Our study in refs (11) and (12) employed Turbomole,^15,16^ which until recently relied on the Maximoff–Scuseria (MS) approach^17^ to turn the kinetic energy density τ used in meta-generalized gradient approximation (mGGA) functionals into a gauge-independent quantity in the presence of a magnetic field. However, recent work by some of us has shown that the MS approach that is also used in the Gaussian^18^ program may lead to artifacts for nuclear magnetic shielding constants, such as artificial paramagnetic contributions to the shielding constant of spherical atoms, which can be avoided by employing Dobson’s current-density extension of τ,^19^ instead;^20−23^ this approach is nowadays available in Turbomole for time-dependent density functional theory (TDDFT) calculations^24−26^ and other properties.^27,28^ The approach is also used in other packages as well for TDDFT and NMR calculations.^29,30^ We furthermore note that Dobson’s current-density extension for τ has been found to be important in the context of calculations in explicit magnetic fields.^31−35^

Holzer et al.^27^ recently employed the methodology of refs (11) and (12) to compare the effect of the MS and Dobson formulations of τ on the magnetizabilities of two test sets: the aforementioned theory-based test set of ref (7) and a test set based on experimental data. Their results appeared, at first sight, somewhat inconclusive as the two test sets resulted in different rankings of various functionals.

Several local hybrid (LH) functionals that employ a position-dependent exact-exchange (EXX) admixture^36,37^ and that have been shown to exhibit promising accuracy for nuclear magnetic shielding constants^20−23^ performed excellently for the theory-based test set, with relative mean absolute deviations in magnetizabilities below 1%. However, Holzer et al.^27^ put more emphasis on the comparison to experimental data, in which deviations for most functionals including LH functionals were generally above 5% and were less systematic than in the theory-based benchmark.

We argue that the comparison to these experimental data does not afford a reliable assessment of the accuracy of density functionals, as many of these data exhibit large error bars. As LH functionals are of interest for many kinds of properties, we revisit their performance for magnetizabilities in this work, based on the theoretical data set of Lutnæs et al.,^7^ which was also used in previous studies.^11,12,27^ Although the limitations of this kind of static DFT benchmarks need to be recognized,^38^ such studies often give helpful guidance in terms of the suitable physical contents of density functional approximations.

Going beyond the LH functionals studied in ref (27), we also include the first modern range-separated local hybrid (RSLH) ωLH22t^39^ in our assessment, as it has been shown to provide remarkable accuracy for quasiparticle energies of a wide variety of organic chromophores of interest in molecular electronics and organic photovoltaics,^40^ while also performing well for many other ground- and excited-state properties.^39^ We also investigate the performance of recent strong-correlation-corrected LH functionals (scLH) such as scLH22t and scLH22ta,^41^ as well as the most recent models with simplified constructions of the sc-correction terms.^42^ We will show that these functionals can more reliably reproduce magnetizabilities of systems with large static correlation effects such as O_3_, whose magnetizabilities predicted by regular LH functionals significantly deviate from the CCSD(T) reference value.^27^

In addition, we will also study the Dobson formulation for τ on a wider set of τ-dependent functionals than those studied in ref (27), including various older and newer mGGA functionals and mGGA-based global and RSH functionals.

The layout of this work is as follows. Next, in Section 2, we discuss the employed methodology for computing the magnetizability (Section 2.1), gauge-origin problems (Section 2.2), and LH functionals (Section 2.3) and then the employed computational methodology in Section 3. We present the results of this study in Section 4, and we conclude in Section 5. Atomic units are used throughout, unless specified otherwise.

## 2 Theory

### 2.1 Methods for Calculating Magnetizabilities

Magnetizabilities are commonly calculated as the second derivative of the electronic energy with respect to the external magnetic field^9,43−46^
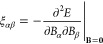
1The magnetic interaction can also be expressed as an integral over the magnetic interaction energy density ρ^**B**^(**r**), which is the scalar product of the magnetically induced current density **J**^**B**^(**r**) with the vector potential **A**^**B**^(**r**) of the external magnetic field **B**(11,12,47−53)
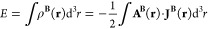
2The second derivatives of the magnetic interaction energy with respect to the components of the external magnetic field, together forming the elements of the magnetizability tensor **ξ**, can be obtained from eq 2 as an integral over the scalar product of the first derivatives of the vector potential of the external magnetic field with the magnetically induced current-density susceptibility (CDT), , in the limit of a vanishing magnetic field^52−54^
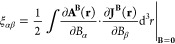
3where the vector potential **A**^**B**^(**r**) of a homogeneous external magnetic field is
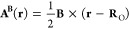
4and **R**_O_ is an arbitrary gauge origin. The CDT can be calculated in a given atomic-orbital (AO) basis set from the unperturbed and the magnetically perturbed AO density matrices, which can be obtained at any level of theory from nuclear magnetic shielding calculations, for example.^55−58^

The isotropic magnetizability  is obtained as one-third of the trace of the magnetizability tensor
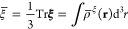
5where the magnetizability density tensor is defined in terms of the CDT as
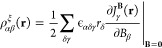
6where ϵ_αδγ_ is the Levi–Civita symbol, and α, β, γ, δ and ∈ {*x*, *y*, *z*} are Cartesian directions. The ξ_αα_, α ∈ {*x*, *y*, *z*} elements of the magnetizability tensor can be obtained by quadrature as
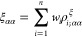
7where ρ_*i*;αα_^ξ^ is a diagonal element of the magnetizability density tensor at quadrature point *i*, and *w*_*i*_ is the corresponding quadrature weight.

The use of the above quadrature scheme allows magnetizabilities to be computed even when the analytical second derivatives required to evaluate eq 1 are not available.^11^ In addition to providing a complementary approach to computing magnetizabilities, the numerical integration approach also affords information about spatial contributions to the magnetizability;^11,12^ similar approaches have previously also been used to study the spatial contributions to nuclear magnetic shielding constants.^59−68^

### 2.2 Gauge-Origin Problems

The use of a finite one-particle basis set introduces issues with gauge dependence into quantum chemical calculations of magnetic properties. The CDT can be made gauge-origin independent^55,57,58,69^ by using gauge-including atomic orbitals (GIAOs),^4,70−73^ also known as London atomic orbitals (LAOs)^43,46,74^
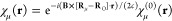
8where *i* is the imaginary unit, χ_μ_^(0)^(**r**) is a basis function centered at **R**_μ_, and *c* is the speed of light which has the value *c* = α^–1^ ≈ 137.036 in atomic units, where α is the fine-structure constant.

The mGGA approximation for the exchange–correlation energy density contains a dependence on the kinetic energy density τ, which reads in the field-free case as
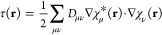
9where *D*_μν_ is the AO density matrix. However, this dependence requires additional care, as the (uncorrected) τ(**r**) of eq 9 is clearly not gauge invariant a priori even when using GIAOs.

A widely used model to render τ(**r**) gauge invariant was introduced by Maximoff and Scuseria^17^ (MS) as
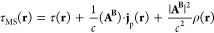
10where **j**_p_(**r**) is the paramagnetic current density defined as
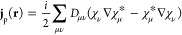
11

Advantages for coupled-perturbed KS (CPKS) calculations with mGGAs arise from the diagonality of the Hessian for τ_MS_(**r**), which allows one to solve the CPKS equations in a single step. However, the semilocal exchange–correlation (XC) contribution does not produce a linear response, even though a genuine current-density functional is expected to provide such a response.^75,76^ τ_MS_(**r**) also does not constitute a proper iso-orbital indicator,^77^ is nonuniversal,^24,33,78^ and introduces paramagnetic artifacts in shielding calculations.^20^

A formulation that avoids the disadvantages of the MS model was proposed by Dobson^19,79^
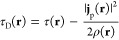
12τ_D_(**r**) in eq 12 leads to gauge independence while also introducing a current response of the semilocal XC contribution and thereby a correct physical behavior for τ-dependent density functionals. As the electronic Hessian corresponding to eq 12 is nondiagonal, an iterative solution to the CPKS equations is now necessary, rendering the calculations slightly more expensive than when the physically incorrect eq 10 is used.

### 2.3 Local Hybrid Functionals

In addition to studying the importance of the Dobson formulation (eq 12) of the kinetic-energy density in reproducing accurate magnetizabilities, we also evaluate the accuracy of LH functionals. A form for an LH functional that emphasizes the inclusion of nonlocal correlation terms (often considered to cover nondynamical correlation, NDC) together with full exact exchange and a (semilocal) dynamical correlation (DC) functional is^13,37^
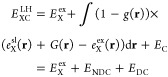
13where *g*(**r**) is the local mixing function (LMF) controlling the fraction of exact exchange included at **r**. In most of the LH functionals considered here, we use a so-called t-LMF defined as the scaled ratio between the von Weizsäcker^80^ and KS kinetic energy densities
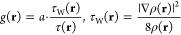
14

The calibration function (CF) *G*(**r**) is used in eq 13 to correct for the ambiguity of the semilocal and exact exchange-energy densities.^37,81−84^ In the LH functionals with a CF from the Berlin group,^39,41,85^ the semilocal CFs are currently derived within the partial integration gauge (pig) approach.^83^

We also evaluate two recent extensions of the LH functionals: the so-called strong correlation (sc) LH functionals^41,42,86^ and a RSLH functional (ωLH22t).^39^ In the scLH functionals, a strong-correlation factor *q*_AC_(**r**) is introduced into the LMF of the LH
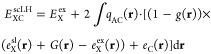
15

This approach is based on the local adiabatic connection approach and is adapted from the KP16/B13^87^ and B13^88^ sc-models. The most recent models employ simplified real-space measures to detect strong correlations, as well as modified damping functions to avoid double-counting of NDC contributions in more weakly correlated situations.^42^

In the absence of strong correlations, *q*_AC_ → 0.5 is a lower bound, and the underlying LH functional is restored. Whenever the quantities underlying *q*_AC_ detect locally the presence of strong correlations, *q*_AC_ is increased maximally up to 1.0. In the exchange picture, this means that the EXX admixture is locally diminished; in some cases it may even become negative.^41,42^ This enhances the simulation of nonlocal correlation contributions and is crucial for reducing fractional spin errors and for improving spin-restricted bond dissociation curves.^41,42^

RSLH functionals like ωLH22t^39^ may be written as
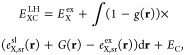
16where *e*_X,sr_^sl^ and *e*_X,sr_^ex^ are short-range exchange-energy densities, controlled by the range–separation parameter ω. In consequence, the ωLH22t functional has a full long-range EXX admixture,^39^ like the RSH functionals evaluated here as well, while the short-range EXX admixture is determined by the LMF.

## 3 Computational Methods

As in refs (11) and (12), the unperturbed and magnetically perturbed density matrices are generated with the nuclear magnetic shielding module of the Turbomole program (mpshift).^16^ A locally modified version of Turbomole 7.6 was used for the present calculations. Turbomole employs LIBXC^89^ to evaluate many of the presently considered density functionals. A detailed description of the implementations of the LH functionals,^20^ the Dobson model,^23^ and higher derivatives of the density used in the pig2 CF^21^ can be found in the respective publications. The necessary equations for the magnetic-field derivatives of scLHs and RSLHs are outlined in Section S1 of the Supporting Information.

The calculations were carried out with the aug-cc-pCVQZ basis set^90−95^ (with aug-cc-pVQZ on hydrogen atoms) employing GIAOs,^71,72,74,96^ a.k.a. LAOs.^43,46^ The self-consistent field convergence criteria were set as 10^–9^ for the energy and 10^–7^ for the density. Large numerical integration grids per the approach of Becke^97^ were used with the Turbomole^15,16^ setting grid size 7, following the original work of Treutler and Ahlrichs^98^ with later extensions documented in the Turbomole manual.^99^ The nonstandard exact-exchange integrals occurring in LH, scLH, and RSLH functionals are calculated by efficient seminumerical integration techniques^100−103^ using standard DFT grids.

The resolution-of-the-identity (RI) approximation was used to evaluate the Coulomb contribution (J), using Turbomole’s “universal” auxiliary basis set by Weigend.^104^ Although one needs to be careful about mixing auxiliary basis sets for different families, we tested the accuracy of this RI-J approximation with a few of the functionals considered in this work, and the resulting magnetizabilities with the RI-J approximation coincided with values obtained without it to the reported number of digits, which is consistent with results recently computed for NMR shieldings.^105,106^ We note that accurate auxiliary basis sets with controllable accuracy for RI calculations can nowadays be easily generated automatically^107,108^ and recommend such autogenerated auxiliary basis sets to be used when tailored basis sets are not available. We refer the reader to ref (109) for a recent review of further automatic auxiliary basis generation techniques.

The magnetizabilities are computed from the density matrices produced by Turbomole with the GIMIC program.^55−58^ The integral in eq 6 is calculated in GIMIC using Becke’s^97^ multicenter quadrature scheme,^11,12^ employing the Numgrid library^110^ to generate the atomic quadrature grids with a hardness parameter of 3 for the atomic weight partitioning, radial grids from Lindh et al.,^111^ and Lebedev’s angular grids.^112^ Both Gimic and Numgrid are free and open-source software.^113,114^

In this work, we study the accuracy of magnetizabilities reproduced by the 31 functionals listed in Table 1 for a data set of 28 molecules: AlF, C_2_H_4_, C_3_H_4_, CH_2_O, CH_3_F, CH_4_, CO, FCCH, FCN, H_2_C_2_O, H_2_O, H_2_S, H_4_C_2_O, HCN, HCP, HF, HFCO, HOF, LiF, LiH, N_2_, N_2_O, NH_3_, O_3_, OCS, OF_2_, PN, and SO_2_, which have also been used as benchmark molecules in other studies.^7,11,12^ The obtained DFT magnetizabilities are compared to the CCSD(T) reference values of ref (7), from where we also took the employed molecular geometries. Since the magnetizability for O_3_ introduces significant uncertainties due to large static correlation effects, it was omitted from the statistical analysis, as was also done in refs (11) and (12).

**Table 1:** Employed Local Hybrid (LH) Functionals, Range-Separated LH (RSLF) Functionals, Strong Correlation LH (scLH) Functionals, Functionals at the Meta-Generalized Gradient Approximation (mGGA), Global Hybrid (GH) Functionals as Well as Range-Separated (RS) GGA and mGGA Functionals, and One GGA Functional

functional	type	notes	references
scLH23t-mBR^a^	scLH	ct-LMF (*a* ≈ 0.715), damped *q*_AC-erf_, pig2-CF, X_0.22S+0.78PBE_ + C_modB95_	(42)
scLH23t-mBR-P^a^	scLH	ct-LMF (*a* ≈ 0.715), damped , pig2-CF, X_0.22S+0.78PBE_ + C_modB95_	(42)
scLH22t	scLH	ct-LMF (*a* = 0.715), damped *q*_AC_, pig2-CF, X_0.22S+0.78PBE_ + C_modB95_	(41)
scLH22ta	scLH	ct-LMF (*a* = 0.766), *q*_AC_, pig2-CF, X_0.04S+0.96PBE_ + C_modB95_	(41)
scLH21ct-SVWN-m	scLH	ct-LMF (*a* = 0.628), *q*_AC_, X_S_ + C_VWN_	(86)
ωLH22t	RSLH	ct-LMF (*a* = 0.587), ω = 0.233, pig2-CF, X _PBE_ + C_modB95_	(39)
LH20t	LH	ct-LMF (*a* = 0.715), pig2-CF, X_0.22S+0.78PBE_ + C_modB95_	(85)
LH20t nonCal	LH	ct-LMF (*a* = 0.715), X_0.22S+0.78PBE_ + C_modB95_	(85)
LH14t-calPBE	LH	t-LMF (*a* = 0.5), pig1-CF, X_0.49S+0.51PBE_ + C_0.55PW92+0.45PBE_	(115)
LH12ct-SsirPW92	LH	ct-LMF (*a* = 0.646), X_S_ + C_sicPW92_	(116)
LH12ct-SsifPW92	LH	ct-LMF (*a* = 0.709), X_S_ + C_sicPW92_	(116)
LH07t-SVWN	LH	t-LMF (*a* = 0.48), X_S_ + C_VWN_	(117,118)
LH07s-SVWN	LH	s-LMF (*b* = 0.277), X_S_ + C_VWN_	(119)
mPSTS-a1^b^	LH	modified PSTS functional	(27,120)
mPSTS-noa2^b^	LH	modified PSTS functional	(27,120)
LHJ14	LH	z-LMF (*c* = 0.096), X_B88_ + C_B88_	(121)
B97M-V	mGGA		(122)
VSXC	mGGA		(123)
MN15-L	mGGA		(124)
TPSS	mGGA		(125,126)
M06-L	mGGA		(127)
τ-HCTH	mGGA		(128)
PW6B95	GH, mGGA		(129)
TPSSh	GH, mGGA		(125,126)
M06-2X	GH, mGGA		(130)
M06	GH, mGGA		(130)
MN15	GH, mGGA		(131)
B3LYP5^c^	GH, GGA		(132,133)
BHandHLYP	GH, GGA		(134)
ωB97M-V	RSH, mGGA		(135)
ωB97X-V	RSH, GGA		(136)

a Use of simplified *q*_AC_ based on error function (scLH23t-mBR) or Padé functions (scLH23t-mBR-P); simplified construction of underlying function to identify regions of strong-correlation; see ref (42) for details.

b See ref (27) for further details of the a1 and noa2 models.

c This functional is called B3LYP in Turbomole, and as discussed by Hertwig and Koch,^133^ it is slightly different from the B3LYP functional used in ref (11).

## 4 Results

We will begin the discussion of the results in Section 4.1 by examining the accuracy of all of the considered functionals with the Dobson and MS models for τ in the subset of data without O_3_, which exhibits strong correlation effects. We then discuss the accuracy of the various functionals of O_3_ in Section 4.2. The magnetizabilities for all studied molecules and functionals with the MS and Dobson formulations of τ can be found in the Supporting Information, accompanied by violin plots of the corresponding error distributions.

### 4.1 Accuracy of Density Functionals with Various Models for τ

Table 2 summarizes the overall statistical evaluations and the rankings of the various functionals within the Dobson and MS models for τ. We begin by noting that our results agree with those of Holzer et al.^27^ for the subset of functionals also studied in ref (27).

**Table 2:** Mean Absolute Errors (MAEs), Mean Errors (MEs), and Standard Deviations (STDs) of the Errors in the Magnetizabilities of the 28 Studied Molecules in Units of 10^–30^ J/T^2^ from the CCSD(T) Reference Values with the Studied Functionals

rank	functional	MAE	ME	STD	rank	MAE	ME	STD
τ_D_					τ_MS_			
1	scLH23t-mBR	2.25	–0.31	3.40	1	2.39	0.36	3.78
2	scLH22t	2.35	–0.29	3.27	2	2.47	0.47	3.62
3	LH20t	2.48	0.46	3.71	4	2.71	1.15	4.09
4	scLH23t-mBR-P	2.52	–0.41	3.81	3	2.69	0.27	4.20
5	scLH22ta	2.67	0.24	3.64	5	2.83	0.66	3.78
6	LH14t-calPBE	3.02	1.28	4.06	8	3.21	2.06	4.21
7	ωLH22t	3.09	0.12	4.06	10	3.49	1.16	4.73
8	BHandHLYP	3.13	2.17	4.61	7	3.13	2.17	4.61
9	ωB97X-V	3.23	2.53	4.31	9	3.23	2.53	4.31
10	LH20t nonCal	3.31	0.23	4.39	14	3.68	1.74	4.97
11	LH07t-SVWN	3.55	0.18	4.46	13	3.68	2.01	4.72
12	scLH21ct-SVWN-m	3.61	–2.73	3.46	6	3.07	–0.15	3.99
13	LH12ct-SsirPW92	3.74	–1.89	4.39	11	3.59	0.14	4.63
14	ωB97M-V	3.87	1.40	5.03	12	3.62	0.43	4.70
15	LH12ct-SsifPW92	4.25	–2.70	4.67	15	3.93	–0.67	4.94
16	B97M-V	4.78	3.50	5.79	16	5.19	4.13	5.48
17	B3LYP5	5.44	4.55	5.93	17	5.44	4.55	5.93
18	LH07s-SVWN	5.84	3.12	7.24	18	5.84	3.12	7.24
19	MN15	6.00	5.02	6.60	29	11.47	10.46	12.63
20	TPSSh	6.82	6.73	6.22	23	7.22	7.09	5.97
21	mPSTS-noa2	6.85	6.83	6.27	22	7.15	7.13	5.99
22	VSXC	6.96	5.24	7.75	20	7.07	5.50	7.52
23	LHJ14	6.98	5.62	6.96	21	7.13	5.56	7.55
24	MN15-L	7.07	–6.84	6.04	19	6.55	–5.25	6.81
25	mPSTS-a1	7.10	7.04	6.34	24	7.42	7.37	6.07
26	PW6B95	7.60	7.26	7.20	26	8.32	8.00	8.10
27	M06-2X	7.80	7.26	9.27	28	10.18	9.03	12.93
28	TPSS	7.83	7.46	6.94	25	8.24	7.88	6.77
29	τ-HCTH	10.20	9.68	8.06	27	9.74	9.22	7.83
30	M06-L	12.93	12.86	11.32	30	12.53	12.49	9.30
31	M06	15.06	14.78	13.12	31	13.37	13.14	12.98

In the mean absolute error (MAE) analysis, the largest effects of using τ_D_ instead of τ_MS_ are obtained for some Minnesota functionals, e.g., – 5.47 × 10^–30^ J/T^2^ for MN15, – 2.38 × 10^–30^ J/T^2^ for M06-2X, and +1.69 × 10^–30^ J/T^2^ for M06. Other functionals that exhibit large (more than 0.5 × 10^–30^ J/T^2^) effects on the MAEs include PW6B95 (−0.72 × 10^–30^ J/T^2^) and MN15-L (+0.52 × 10^–30^ J/T^2^). We note here that many Minnesota functionals, including M06 and M06-2X, have been recently found to be numerically ill-behaved, while MN15 and MN15-L appear to behave better.^137,138^

The MAE values suggest that ensuring proper gauge invariance may either improve or worsen the agreement with the CCSD(T) reference data. Some of us have found a similar behavior for NMR chemical shifts^22,23^ and attributed it to massive error compensation in some of the cases.

However, in most cases, the differences between the MS and Dobson formulations are small, and the rankings of the best-performing functionals are not affected very much by switching from using the MS expression to the Dobson expression. Some exceptions do occur; for instance, the change of −0.40 × 10^–30^ J/T^2^ going from τ_MS_ to τ_D_ leads to an improved ranking by three positions for ωLH22t.

Interesting differences can be seen between the behavior of some first-generation LH functionals based on LSDA exchange-energy densities (e.g., LH12ct-SsirPW92, LH12ct-SsifPW92, and the related scLH21ct-SVWN-m) and that of the more advanced functionals (LH, scLH, and the ωLH22t RSLH functional). The accuracy of functionals of the former type deteriorates somewhat after switching from the computationally convenient τ_MS_ to the physically more correct τ_D_, while the accuracy of the latter type of functionals improves when τ_D_ is used.

A closer analysis of the origin of the differences between the MS and Dobson formulations of τ is beyond the scope of this work. However, a recent study of the Dobson-based gauge-invariance contributions to TDDFT excitation energies has been able to link the magnitude and even the sign of the effect to the way in which τ enters the enhancement factor of the mGGA functionals and other τ-dependent functionals.^26^

LH20t and its sc-corrected extensions scLH22t and scLH23t-mBR occupy ranks 3, 2, and 1, respectively, with the scLHs improving slightly over their parent LH functional. The reduced MAE of scLH23t-mBR and scLH22t as compared to LH20t arises from some of the molecules that are expected to exhibit larger static correlation effects, as evidenced by larger deviations between CCSD(T) and CCSD results in ref (7). Apart from the true static correlation case O_3_ (see below), scLH22t gives notable improvements of more than 2 × 10^–30^ J/T^2^ for PN and SO_2_. scLH23t-mBR gives large improvements for PN and HCP. The performance of both functionals would be even more impressive if not for the somewhat larger deviations of −6.4 × 10^–30^ J/T^2^ (scLH22t) and −5.3 × 10^–30^ J/T^2^ for the LiH molecule as compared to the LH20t value of +3.2 × 10^–30^ J/T^2^. scLH23t-mBR-P, with its Padé-based *q*_AC_, also improves particularly on PN and HCP but deteriorates on LiH (−10.6 × 10^–30^ J/T^2^), hampering the functional’s overall statistical performance compared to the other two scLHs and leaves it slightly behind LH20t in the overall ranking. Effects of the sc-corrections are much smaller for the other molecules, which is consistent with efficient damping of the sc-factor for weakly correlated systems. It is presently unclear why the effects of the sc-corrections are below 1 × 10^–30^ J/T^2^ for several other systems with larger differences between CCSD and CCSD(T) (H_2_CO, OF_2_, and HOF).

The two best-performing functionals in refs (11) and (12) (BHandHLYP and ωB97X-V) are ranked eighth and ninth when considering the Dobson formulation of τ. Strikingly, several LH and scLH functionals, as well as the ωLH22t RSLH functional occupy the seven top positions in the ranking, and several further LH functionals follow in the top 15 of the ranking (Table 2). A similar trend was observed earlier when considering a smaller selection of LH functionals.^27^ However, somewhat different conclusions were then reached because the experimental data used as reference values in that work exhibit very large error bars and are therefore ill-suited for benchmarking purposes.

Individual changes from LH20t to scLH22ta which lack the damping factor are overall somewhat more pronounced and lead to a slightly larger MAE for the scLH functionals, indicating some deterioration of the accuracy of the magnetizability for more weakly correlated systems, which places scLH22ta behind LH20t, but scLH22ta is still ranked fifth best.

**Figure 1:**
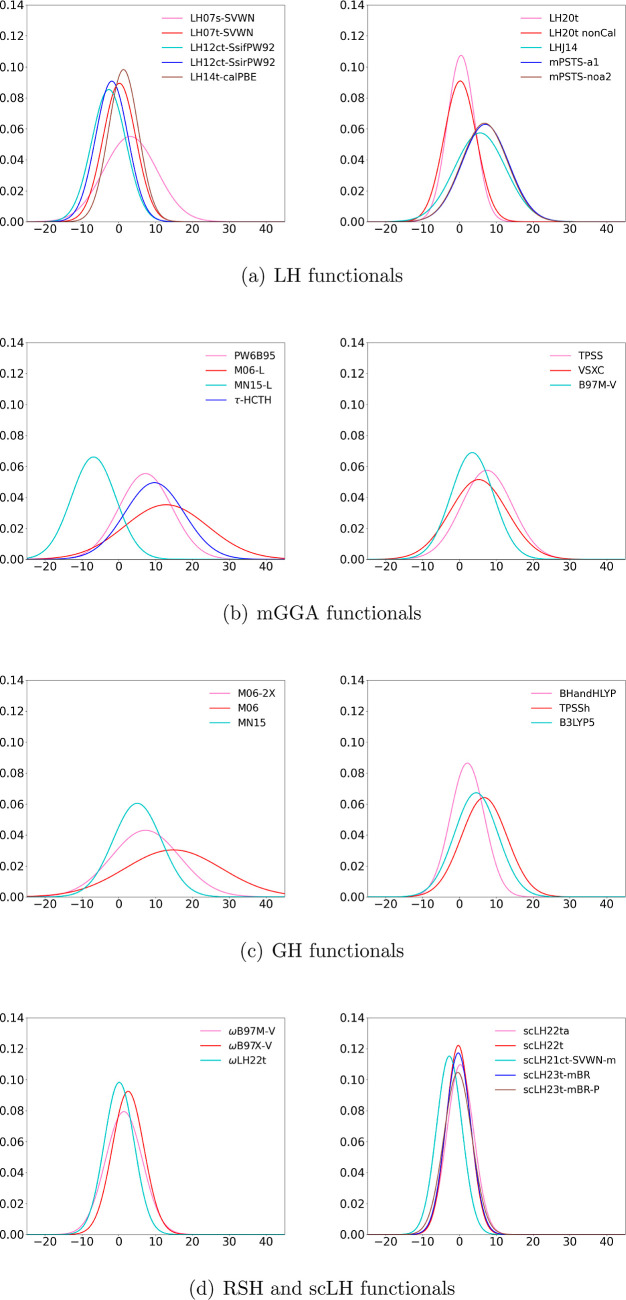
Normal distributions of the magnetizability data calculated with the Dobson formulation of τ.

Further, top-performing functionals include LH14t-calPBE (rank 6) and ωLH22t RSLH (rank 7), but their MAE in the 3.0–3.3 × 10^–30^ J/T^2^ range is already comparable to those of the best-performing functionals from refs (11) and (12) (BHandHLYP, ωB97X-V, and CAM-QTP functionals). In agreement with refs (11) and (12), B97M-V remains the highest-ranked nonhybrid functional in the evaluations, its MAE is reduced from 5.19 × 10^–30^ to 4.78 × 10^–30^ J/T^2^ by using the Dobson formulation of τ. The normal distributions of the magnetizabilities of the studied functionals with the Dobson formula for τ are shown in Figure 1; analogous plots for the MS formula for τ are given in the Supporting Information, along with violin plots of the errors for both descriptions, both with and without the inclusion of O_3_ in the data set.

### 4.2 Accuracy on O_3_

As was already discussed above, due to its large static correlation effects, O_3_ has been excluded from statistical evaluations in previous density functional assessments.^7,11,12^ Its large deviations would otherwise dominate the statistics and it is unclear whether the CCSD(T) reference value is sufficiently accurate for this molecule, given that the inclusion of the perturbational triples (T) contributions reduces the magnetizability by more than 45 × 10^–30^ J/T^2^ as compared to the CCSD value.^7^ In the absence of experimental data, we may estimate a reasonable range of values by also considering earlier complete active space self-consistent field (CASSCF), GIAO (97.8 × 10^–30^ J/T^2^),^139^ and multiconfigurational (MC) individual gauge for localized orbitals (IGLO) (89.7 × 10^–30^ J/T^2^)^140^ results. These values are somewhat smaller than the CCSD(T) reference value of 121.5 × 10^–30^ J/T^2^ and suggest that magnetizabilities around 100 × 10^–30^ J/T^2^ with an error margin of about ±20 × 10^–30^ J/T^2^ seem to define the most likely range. We review the various DFT results in light of this range in Table 3.

**Table 3:** Calculated Isotropic Magnetizabilities [10^–30^ J/T^2^] for Ozone with Various Methods

functional	type	ξ̅ [O_3_]	
		τ_D_	τ_MS_
B3LYP	GH, GGA	238.5	
BHandHLYP	GH, GGA	336.6	
B97M-V	mGGA	189.8	99.3
M06-L	mGGA	244.2	156.2
MN15-L	mGGA	193.7	63.6
PW6B95	mGGA	261.4	288.3
τ-HCTH	mGGA	178.3	177.0
TPSS	mGGA	173.5	151.1
VSXC	mGGA	193.9	155.3
M06	GH, mGGA	417.8	413.4
MN15	GH, mGGA	259.5	570.8
M06-2X	GH, mGGA	328.1	492.9
TPSSh	GH, mGGA	200.1	176.9
ωB97X-V	RSH, GGA	251.2	
ωB97M-V	RSH, mGGA	239.8	225.3
LH07s-SVWN	LH	256.0	
LH07t-SVWN	LH	211.2	207.3
LH12ct-SsifPW92	LH	221.6	227.4
LH12ct-SsirPW92	LH	218.2	220.2
LH14t-calPBE	LH	207.5	207.2
LH20t	LH	211.4	223.1
LH20t nonCal	LH	217.3	229.9
LHJ14	LH	213.6	244.3
mPSTS-a1	LH	198.9	175.5
mPSTS-noa2	LH	207.5	183.8
scLH22ct-SVWN-m	scLH	112.9	90.8
scLH22ta	scLH	101.0	90.8
scLH22t	scLH	112.2	109.5
scLH23t-mBR	scLH	120.4	121.4
scLH23t-mBR-P	scLH	134.4	136.5
ωLH22t	RSLH	224.9	240.9
CCSD(T)-GIAO^a^	WFT	121.5	
CASSCF-GIAO^b^	WFT	97.8	
MC-IGLO^c^	WFT	89.7	

a Ref (11).

b Ref (139).

c Ref (140).

Most functionals overestimate the magnetizability of O_3_ dramatically. Among the standard functionals studied here and in refs (11) and (12), the specialized GGA functionals like KT1, KT2, and KT3 get closest to the reference value, with KT1 providing the smallest value of 131.9 × 10^–30^ J/T^2^. These DFAs are not good performers in general according to the previous magnetizability benchmark^11,12^ and would enter at ranks 19, 21, and 31 with MAE of 5.87 × 10^–30^, 6.42 × 10^–30^, and 9.19 × 10^–30^ J/T^2^, respectively, in the statistical evaluation of Table 2. Other standard GGA functionals like BLYP or BP86 give magnetizabilities of O_3_ around 180 × 10^–30^ J/T^2^.^11^ Similar results for simple GGA functionals were also obtained in the original evaluation of Lutnæs et al.^7^ However, these functionals perform poorly for the entire test set, which is why we do not discuss them further here. Some mGGA functionals like M06-L and B97M-V also attain closer agreement when the τ_MS_ prescription is used, but using the more appropriate τ_D_ results in larger magnetizability and a poorer agreement. When the Dobson formulation is used, most mGGA functionals perform comparably to the simpler GGA functionals, with TPSS and τ-HCTH giving the lowest values of 173.5 × 10^–30^ and 178.5 × 10^–30^ J/T^2^, respectively.

Including exact exchange in GH and RSH functionals significantly increases the calculated magnetizability of ozone and thereby deteriorates the agreement with the reference value; in some cases, the predicted magnetizability is more than 300 × 10^–30^ J/T^2^ (BHandHLYP, M06, and M06-2X). We find a similar trend for all LH functionals without sc-corrections as well as for the ωLH22t RSLH functional that yields values larger than 200 × 10^–30^ J/T^2^. The strikingly good performance of various scLH functionals is thus particularly notable: scLH22ta without damping factor in the sc-corrections gives the lowest value; results for the other sc-functionals are in the range of 112–135 × 10^–30^ J/T^2^.

Given that four of the five scLHs are also among the five best-performing functionals for the entire test set, these results are significant in suggesting that the scLH functionals to some extent indeed escape the usual zero-sum game between delocalization errors and strong-correlation errors, where larger EXX admixtures improve on the former but deteriorate the latter.^141^ Such an “escape” has been found recently for fractional spin errors and the related spin-restricted dissociation curves of diatomics.^41,42^ It is gratifying to see this here for a very different property.

## 5 Conclusions

This work extends in two directions: previous studies of DFT functionals for the computation of molecular magnetizabilities. We considered using a gauge-independent local kinetic energy τ ingredient in a wide variety of mGGA functionals within Dobson’s current-DFT formalism (τ_D_), which has not been considered in as much detail so far. We examined the effects of the Dobson formalism by comparing the obtained magnetizabilities to values calculated with the MS formulation (τ_MS_) used in previous works. We also extended the assessment to LH functionals, i.e., with position-dependent EXX admixtures, in particular to their recent strong-correlation corrected and range-separated variants.

Regarding gauge-invariant formulations of τ, we find that going from the previously used, computationally convenient τ_MS_ to the more physically correct τ_D_ leads to dramatic changes in the magnetizability in some cases, while in other cases the differences between τ_D_ and τ_MS_ are small. The largest effects are seen for some of the highly parametrized mGGA and mGGA hybrid functionals from the Minnesota group, whose numerical behavior has also been recently investigated and found wanting for many functionals.^137,138^

While τ_D_ leads to improved agreement with the CCSD(T) reference data for some functionals, it also deteriorates the agreement for other functionals. Notably, the effects of making τ gauge invariant by the Dobson procedure tend to be smaller for the overall better-performing functionals, which include many LH and RSH functionals.

The overall statistical evaluation of a wide variety of different functionals provided evidence that LH functionals can yield particularly accurate magnetizabilities. Indeed, the seven best-performing functionals in this evaluation are newer LH functionals, their strong-correlation corrected variants, and the recently reported RSLH functional ωLH22t. The overall statistical improvement compared to the so far best-performing functionals is moderate but notable, e.g., MAEs of 2.25 × 10^–30^ J/T^2^, 2.35 × 10^–30^ J/T^2^, and 2.48 × 10^–30^ J/T^2^ for scLH23t-mBR, scLH22t, and LH20t, respectively, compared to 3.11 × 10^–30^ J/T^2^ for BHandHLYP and 3.23 × 10^–30^ J/T^2^ for ωB97X-V.

The most striking result is the dramatic improvement obtained with several of the sc-corrected LH functionals for the static-correlation case O_3_. Importantly, this improvement is achieved while retaining the overall highest accuracy for the weakly correlated systems relevant to the statistical evaluations. This observation, for a totally different property than evaluated so far for such functionals, is a further indication that sc-corrected LH functionals offer an escape from the usual zero-sum game between achieving low fractional charge errors and low fractional spin errors.

## Supporting Information Available

Magnetizabilities, normal distributions, and violin plots of the error distributions of the magnetizabilities calculated with the MS and Dobson expressions of τ and equations necessary to implement the magnetic field derivatives for scLHs and RSLHs (PDF)
