# Supplementary material for: Revisiting Gauge-Independent Kinetic Energy Densities in Meta-GGAs and Local Hybrid Calculations of Magnetizabilities
Source: J Phys Chem A. 2023 Dec 15;127(51):10896–907. doi: 10.1021/acs.jpca.3c06244 (PMC10758120; doi:10.1021/acs.jpca.3c06244)
Supplement: Supplementary file 1 — jp3c06244_si_001.pdf [file jp3c06244_si_001.pdf]

Supporting Information: Revisiting Gauge-Independent Kinetic Energy Densities in Meta-GGAs and Local Hybrid Calculations of Magnetizabilities

Caspar J. Schattenberg,† Artur Wodyński,† Hugo Åström,‡ Dage Sundholm,‡ Martin Kaupp,*,† and Susi Lehtola*,‡,¶

†*Technische Universität Berlin, Institut für Chemie, Theoretische Chemie/Quantenchemie, Sekr. C7, Straße des 17. Juni 135, D-10623, Berlin, Germany*

‡*University of Helsinki, Department of Chemistry, Faculty of Science, P.O. Box 55 (A.I. Virtanens plats 1), FI-00014 University of Helsinki, Finland*

¶*Molecular Sciences Software Institute, Blacksburg, Virginia 24061, United States*

E-mail: martin.kaupp@tu-berlin.de; susi.lehtola@alumni.helsinki.fi

The magnetizabilities determined using the Dobson formulation of τ are given in table S1 for global hybrids (GHs), tables S2 and S3 for local hybrids (LHs), table S4 for meta-GGAs (mGGAs), table S5 for range-separated hybrids (RSHs), and table S6 for strong correlation local hybrids (scLHs). Plots for the normalized error distributions for the Dobson formulation were given in the main text for these functionals. Violin plots of the errors are shown in fig. S1.

The magnetizabilities determined using the Maximoff–Scuseria (MS) formulation of τ are given in table S7 for global hybrids (GHs), tables S8 and S9 for local hybrids (LHs), table S10 for meta-GGAs (mGGAs), table S11 for range-separated hybrids (RSHs), and table S12 for strong correlation local hybrids (scLHs). Plots for the normalized error distributions are given in fig. S2 for the MS formulation. Violin plots of the errors are shown in fig. S3.

A short description of the implementation of magnetic-field derivatives for scLH and RSLH functionals is given in section S1.

Table S1: Magnetizabilities computed with GH functionals using Dobson formulation of τ.

Molecule	M06-2X	M06	MN15	BHandHLYP	TPSSh	B3LYP5	CCSD(T)
AlF	−391.2	−384.3	−401.0	−395.6	−394.3	−396.9	−394.5
C_2H_4	−329.7	−328.6	−334.9	−343.0	−335.3	−336.9	−345.6
C_3H_4	−460.8	−460.6	−464.6	−468.8	−463.3	−463.2	−478.9
CH_2O	−105.5	−104.7	−113.5	−123.8	−119.4	−114.8	−127.4
CH_3F	−316.0	−310.9	−315.5	−314.9	−311.8	−312.4	−315.7
CH_4	−318.1	−314.0	−318.2	−315.7	−313.0	−317.2	−316.9
CO	−198.8	−192.1	−203.3	−205.0	−203.3	−206.8	−209.5
FCCH	−439.2	−432.4	−438.7	−443.6	−438.9	−440.2	−441.6
FCN	−365.5	−357.3	−364.7	−370.5	−366.0	−367.6	−370.0
H_2C_2O	−419.0	−414.9	−421.1	−425.0	−420.4	−422.3	−423.9
H_2O	−235.3	−232.9	−234.8	−234.0	−234.9	−236.8	−235.1
H_2S	−456.0	−450.3	−453.4	−453.5	−451.6	−455.4	−455.1
H_4C_2O	−535.7	−524.0	−529.9	−534.5	−527.3	−527.0	−535.2
HCN	−263.1	−251.3	−266.0	−272.7	−265.3	−269.6	−271.8
HCP	−477.0	−463.2	−485.7	−494.3	−482.9	−487.8	−492.8
HF	−176.5	−175.1	−176.6	−175.8	−177.4	−178.5	−176.4
HFCO	−295.0	−290.4	−296.2	−304.0	−298.9	−300.7	−307.2
HOF	−236.0	−227.2	−234.5	−236.7	−231.4	−231.1	−235.4
LiF	−193.4	−190.5	−195.3	−192.6	−194.6	−194.8	−195.5
LiH	−129.8	−130.9	−132.5	−126.4	−127.5	−131.2	−127.2
N_2	−194.5	−180.6	−197.5	−201.6	−197.8	−202.2	−205.2
N_2O	−333.7	−324.6	−332.7	−336.6	−332.5	−333.9	−339.1
NH_3	−291.0	−287.4	−290.3	−289.3	−288.4	−291.4	−290.3
OCS	−578.5	−569.0	−578.9	−585.6	−576.9	−579.8	−584.1
O_3	328.1	417.8	259.5	336.6	200.1	238.5	121.5
OF_2	−246.7	−226.9	−243.3	−250.2	−234.4	−234.1	−247.1
PN	−276.6	−248.3	−297.4	−295.5	−284.7	−292.5	−308.2
SO_2	−285.3	−272.4	−288.0	−296.1	−290.3	−295.8	−314.3

Table S2: Magnetizabilities computed with LH functionals using Dobson formulation of τ.

Molecule	LH07s-SVWN	LH07t-SVWN	LH12ct-SsifPW92	LH12ct-SsirPW92	CCSD(T)
AlF	−398.9	−399.8	−403.4	−402.3	−394.5
C_2H_4	−336.8	−343.1	−346.5	−345.7	−345.6
C_3H_4	−468.4	−474.6	−479.3	−478.0	−478.9
CH_2O	−112.7	−121.7	−124.0	−124.2	−127.4
CH_3F	−316.4	−318.5	−321.4	−320.5	−315.7
CH_4	−321.6	−321.0	−324.2	−322.8	−316.9
CO	−205.9	−207.9	−208.7	−208.5	−209.5
FCCH	−442.9	−444.8	−447.9	−447.0	−441.6
FCN	−369.4	−371.8	−374.7	−373.9	−370.0
H_2C_2O	−428.1	−430.1	−434.7	−433.2	−423.9
H_2O	−237.6	−237.4	−237.5	−237.4	−235.1
H_2S	−459.4	−460.0	−464.2	−462.5	−455.1
H_4C_2O	−533.5	−537.9	−543.9	−542.0	−535.2
HCN	−268.0	−271.3	−273.3	−272.9	−271.8
HCP	−484.1	−489.6	−492.9	−492.2	−492.8
HF	−178.8	−178.4	−178.0	−178.1	−176.4
HFCO	−301.0	−305.0	−307.3	−306.8	−307.2
HOF	−232.9	−235.9	−238.8	−238.0	−235.4
LiF	−197.5	−197.4	−197.7	−197.6	−195.5
LiH	−134.3	−126.3	−127.6	−126.8	−127.2
N_2	−200.3	−203.4	−204.4	−204.1	−205.2
N_2O	−336.8	−340.5	−343.4	−342.6	−339.1
NH_3	−293.3	−293.4	−294.7	−294.1	−290.3
OCS	−583.1	−587.3	−592.2	−590.9	−584.1
O_3	256.0	211.2	221.6	218.2	121.5
OF_2	−235.8	−242.5	−249.6	−247.8	−247.1
PN	−288.2	−298.0	−302.0	−301.1	−308.2
SO_2	−294.0	−301.6	−304.6	−303.9	−314.3

Table S3: Magnetizabilities computed with LH functionals using Dobson formulation of τ.

Molecule	LH14t-calPBE	LH20t	LH20t nonCal	LHJ14	mPSTS-a1	mPSTS-noa2	CCSD(T)
AlF	−398.3	−399.8	−400.8	−395.0	−393.1	−392.9	−394.5
C_2H_4	−342.5	−344.8	−343.9	−334.4	−334.9	−335.3	−345.6
C_3H_4	−472.2	−473.6	−475.1	−462.9	−463.5	−464.0	−478.9
CH_2O	−122.5	−127.1	−125.5	−111.6	−120.6	−121.7	−127.4
CH_3F	−316.7	−317.2	−318.8	−311.6	−312.0	−312.4	−315.7
CH_4	−318.6	−317.1	−319.6	−318.3	−311.9	−311.4	−316.9
CO	−207.4	−207.6	−206.6	−206.8	−202.7	−202.5	−209.5
FCCH	−443.5	−443.8	−444.2	−438.0	−439.0	−439.5	−441.6
FCN	−370.8	−371.2	−371.4	−366.0	−365.9	−366.2	−370.0
H_2C_2O	−426.8	−425.5	−428.6	−420.5	−420.4	−420.8	−423.9
H_2O	−237.0	−236.8	−236.4	−237.8	−234.4	−234.0	−235.1
H_2S	−457.9	−457.5	−459.9	−454.3	−450.7	−450.3	−455.1
H_4C_2O	−535.4	−537.7	−539.5	−525.2	−527.1	−527.8	−535.2
HCN	−271.3	−272.3	−270.7	−268.1	−264.7	−264.9	−271.8
HCP	−489.9	−491.2	−488.9	−481.7	−482.2	−482.7	−492.8
HF	−178.3	−177.9	−177.4	−179.6	−177.1	−176.7	−176.4
HFCO	−304.2	−305.3	−305.0	−299.3	−298.3	−298.5	−307.2
HOF	−234.9	−236.7	−236.9	−229.8	−231.5	−232.1	−235.4
LiF	−196.7	−196.4	−196.6	−197.0	−195.6	−195.5	−195.5
LiH	−125.1	−124.0	−124.4	−134.4	−126.6	−126.6	−127.2
N_2	−203.1	−203.1	−201.8	−201.9	−197.3	−197.0	−205.2
N_2O	−338.6	−338.5	−339.6	−332.7	−332.8	−333.1	−339.1
NH_3	−292.3	−292.0	−292.4	−292.1	−287.7	−287.3	−290.3
OCS	−585.4	−586.7	−587.9	−576.9	−577.0	−577.7	−584.1
O_3	207.5	211.4	217.3	213.6	198.9	207.5	121.5
OF_2	−241.5	−246.9	−247.4	−229.5	−234.3	−236.3	−247.1
PN	−298.0	−299.9	−297.7	−291.2	−283.8	−283.9	−308.2
SO_2	−300.5	−300.7	−300.6	−295.4	−289.1	−288.6	−314.3

Table S4: Magnetizabilities computed with mGGA functionals using Dobson formulation of τ.

Molecule	PW6B95	M06-L	MN15-L	τ-HCTH	TPSS	VSXC	B97M-V	CCSD(T)
AlF	−393.6	−384.7	−415.4	−393.4	−394.9	−399.0	−399.8	−394.5
C_2H_4	−333.4	−329.1	−350.4	−330.6	−333.8	−335.2	−338.8	−345.6
C_3H_4	−460.9	−464.7	−480.6	−457.0	−461.2	−463.4	−466.3	−478.9
CH_2O	−111.8	−117.0	−132.3	−115.0	−117.5	−112.6	−125.8	−127.4
CH_3F	−312.5	−312.5	−319.4	−308.3	−310.5	−311.2	−312.4	−315.7
CH_4	−316.1	−311.1	−324.2	−313.0	−312.9	−319.4	−316.5	−316.9
CO	−202.8	−192.5	−211.0	−203.7	−204.0	−206.5	−205.7	−209.5
FCCH	−437.6	−435.5	−455.2	−435.3	−438.0	−441.1	−443.3	−441.6
FCN	−364.7	−360.3	−379.8	−362.8	−365.3	−368.2	−370.1	−370.0
H_2C_2O	−419.1	−420.0	−443.5	−418.3	−419.3	−427.1	−428.8	−423.9
H_2O	−234.9	−230.6	−236.7	−233.8	−235.9	−235.8	−234.1	−235.1
H_2S	−453.5	−449.1	−463.7	−449.6	−451.8	−457.0	−455.3	−455.1
H_4C_2O	−527.6	−526.0	−544.6	−518.6	−524.4	−524.3	−528.2	−535.2
HCN	−265.5	−251.5	−276.4	−263.1	−264.6	−266.5	−268.3	−271.8
HCP	−480.4	−464.8	−513.3	−479.0	−481.7	−485.9	−488.9	−492.8
HF	−176.8	−173.9	−177.1	−176.3	−178.4	−177.6	−176.3	−176.4
HFCO	−296.9	−291.9	−312.0	−295.6	−298.1	−300.8	−303.7	−307.2
HOF	−231.4	−229.4	−240.8	−225.2	−229.5	−227.7	−231.9	−235.4
LiF	−193.7	−192.0	−197.7	−192.6	−195.3	−196.2	−195.8	−195.5
LiH	−131.4	−128.2	−138.1	−134.2	−129.0	−135.1	−132.1	−127.2
N_2	−198.0	−182.8	−202.9	−197.0	−198.1	−199.9	−199.5	−205.2
N_2O	−331.6	−329.5	−343.2	−329.0	−331.5	−334.1	−334.9	−339.1
NH_3	−289.5	−283.8	−294.3	−287.4	−289.0	−290.9	−288.6	−290.3
OCS	−576.1	−571.3	−596.2	−571.2	−574.9	−579.1	−581.1	−584.1
O_3	261.4	244.2	193.7	178.3	173.5	193.9	189.8	121.5
OF_2	−236.7	−230.3	−252.3	−221.0	−228.3	−223.6	−237.4	−247.1
PN	−283.4	−255.1	−314.1	−285.3	−284.1	−287.7	−289.1	−308.2
SO_2	−288.0	−279.1	−313.5	−286.4	−290.6	−296.6	−296.9	−314.3

Table S5: Magnetizabilities computed with RSH functionals using Dobson formulation of τ.

Molecule	ωB97M-V	ωB97X-V	ωLH22t	CCSD(T)
AlF	−397.9	−396.4	−398.9	−394.5
C_2H_4	−338.6	−338.9	−343.2	−345.6
C_3H_4	−467.5	−469.9	−474.6	−478.9
CH_2O	−115.1	−118.6	−121.5	−127.4
CH_3F	−314.0	−314.9	−318.1	−315.7
CH_4	−319.9	−317.7	−320.8	−316.9
CO	−210.3	−207.4	−208.3	−209.5
FCCH	−443.6	−442.4	−444.1	−441.6
FCN	−371.2	−370.1	−371.5	−370.0
H_2C_2O	−425.8	−425.3	−427.2	−423.9
H_2O	−237.3	−236.0	−237.6	−235.1
H_2S	−457.2	−454.2	−459.4	−455.1
H_4C_2O	−531.3	−532.8	−538.3	−535.2
HCN	−273.7	−270.7	−272.4	−271.8
HCP	−489.6	−486.5	−488.5	−492.8
HF	−178.7	−178.1	−178.6	−176.4
HFCO	−303.1	−303.1	−304.7	−307.2
HOF	−234.5	−234.7	−236.7	−235.4
LiF	−196.3	−196.4	−197.3	−195.5
LiH	−134.9	−127.4	−127.2	−127.2
N_2	−206.4	−202.9	−204.2	−205.2
N_2O	−336.6	−336.8	−339.1	−339.1
NH_3	−292.4	−290.5	−293.5	−290.3
OCS	−584.8	−584.7	−587.3	−584.1
O_3	239.8	251.2	224.9	121.5
OF_2	−243.9	−243.8	−246.8	−247.1
PN	−299.8	−296.0	−299.3	−308.2
SO_2	−301.7	−299.5	−301.8	−314.3

Table S6: Magnetizabilities computed with scLH functionals using Dobson formulation of τ.

Molecule	scLH22ta	scLH22t	scLH21ct-SVWN-m	scLH23t-mBR	scLH23t-mBR-P	CCSD(T)
AlF	−404.1	−399.9	−405.5	−399.8	−399.8	−394.5
C_2H_4	−341.6	−344.3	−344.7	−344.7	−344.7	−345.6
C_3H_4	−471.1	−472.9	−475.7	−473.7	−473.6	−478.9
CH_2O	−126.4	−127.6	−129.6	−127.0	−127.3	−127.4
CH_3F	−315.4	−317.0	−317.2	−317.2	−317.2	−315.7
CH_4	−317.3	−317.5	−318.6	−317.3	−317.2	−316.9
CO	−210.0	−208.0	−212.2	−207.6	−207.7	−209.5
FCCH	−441.7	−443.9	−446.7	−443.8	−443.8	−441.6
FCN	−370.6	−371.4	−375.2	−371.2	−371.3	−370.0
H_2C_2O	−425.5	−426.1	−432.8	−425.6	−425.7	−423.9
H_2O	−238.8	−236.8	−238.6	−236.8	−236.8	−235.1
H_2S	−458.8	−458.0	−458.7	−458.0	−458.1	−455.1
H_4C_2O	−535.1	−537.3	−536.5	−537.6	−537.6	−535.2
HCN	−270.8	−272.6	−274.1	−272.3	−272.4	−271.8
HCP	−487.5	−492.2	−494.3	−494.1	−493.3	−492.8
HF	−179.8	−177.9	−179.6	−177.9	−177.9	−176.4
HFCO	−307.2	−305.6	−310.6	−305.3	−305.4	−307.2
HOF	−234.6	−236.5	−236.1	−236.6	−236.6	−235.4
LiF	−199.2	−196.5	−200.5	−196.4	−196.4	−195.5
LiH	−128.2	−133.6	−134.2	−132.5	−137.8	−127.2
N_2	−202.8	−203.4	−206.2	−203.1	−203.2	−205.2
N_2O	−338.0	−338.8	−343.3	−338.7	−338.6	−339.1
NH_3	−293.2	−292.1	−293.5	−292.0	−292.0	−290.3
OCS	−585.4	−587.1	−591.0	−586.7	−586.8	−584.1
O_3	101.0	112.2	112.9	120.4	134.4	121.5
OF_2	−240.5	−246.9	−241.2	−246.9	−247.0	−247.1
PN	−302.8	−304.3	−306.3	−308.4	−304.9	−308.2
SO_2	−310.8	−303.7	−315.1	−301.1	−301.7	−314.3

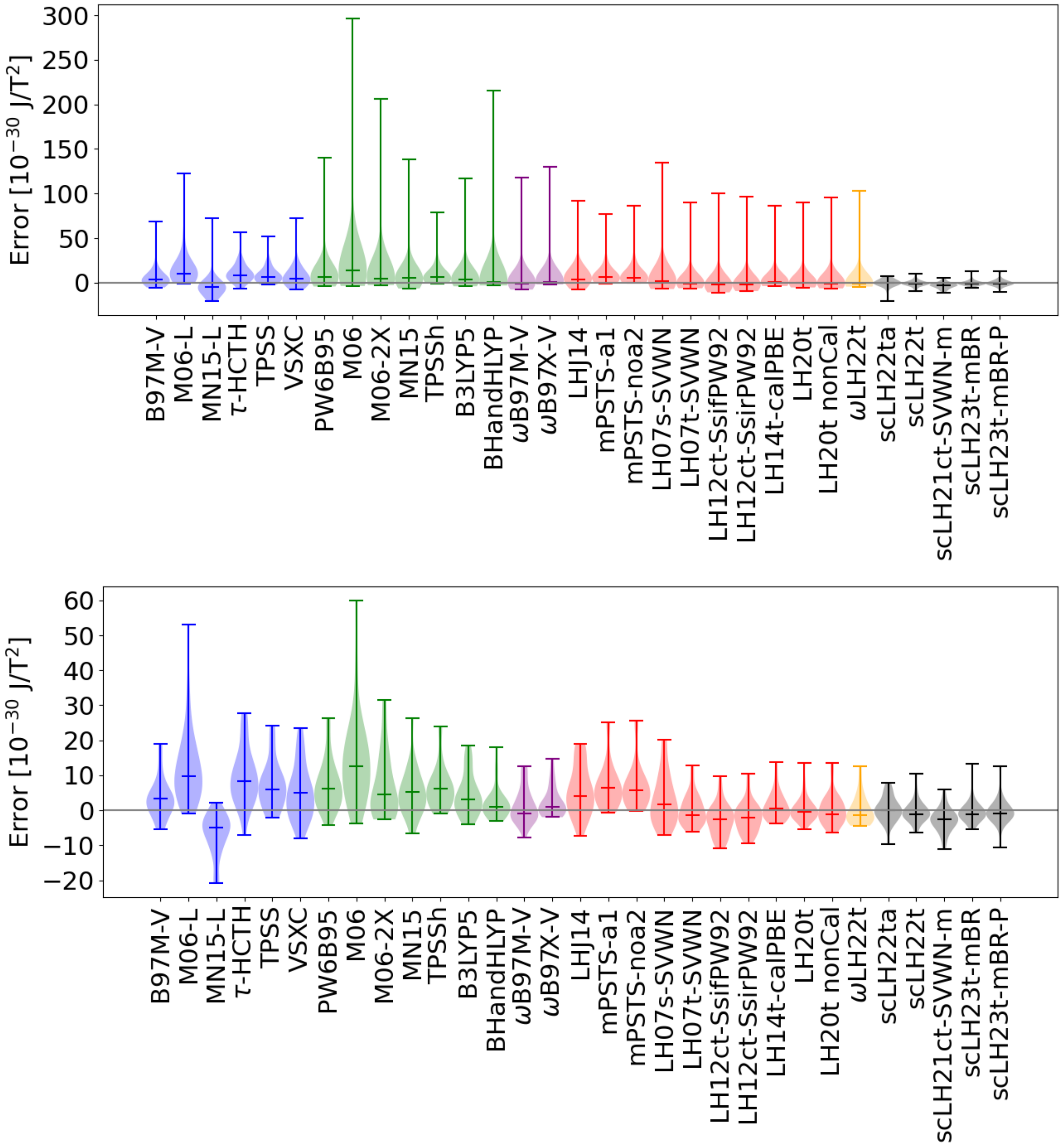

Figure S1: Violin plots of the error distribution with (top) and without (bottom) ozone using the Dobson formulation of τ for meta-GGA functionals.

Table S7: Magnetizabilities computed with GH functionals using MS formulation of τ.

Molecule	M06-2X	M06	MN15	BHandHLYP	TPSSh	B3LYP5	CCSD(T)
AlF	−392.9	−387.1	−400.3	−395.6	−393.3	−396.9	−394.5
C_2H_4	−329.9	−332.4	−330.4	−343.0	−333.8	−336.9	−345.6
C_3H_4	−461.4	−465.3	−460.0	−468.8	−460.9	−463.2	−478.9
CH_2O	−94.2	−104.5	−91.0	−123.8	−121.7	−114.8	−127.4
CH_3F	−317.2	−313.4	−314.9	−314.9	−310.9	−312.4	−315.7
CH_4	−319.9	−316.3	−319.4	−315.7	−311.8	−317.2	−316.9
CO	−193.9	−192.3	−194.3	−205.0	−203.9	−206.8	−209.5
FCCH	−439.7	−434.5	−437.2	−443.6	−437.8	−440.2	−441.6
FCN	−364.6	−358.7	−361.1	−370.5	−365.5	−367.6	−370.0
H_2C_2O	−418.1	−417.3	−416.3	−425.0	−419.1	−422.3	−423.9
H_2O	−235.6	−233.1	−235.1	−234.0	−234.7	−236.8	−235.1
H_2S	−457.7	−451.7	−454.8	−453.5	−450.5	−455.4	−455.1
H_4C_2O	−540.3	−529.4	−531.5	−534.5	−524.5	−527.0	−535.2
HCN	−260.4	−252.4	−259.8	−272.7	−265.1	−269.6	−271.8
HCP	−476.4	−465.0	−481.5	−494.3	−481.9	−487.8	−492.8
HF	−176.6	−175.1	−176.8	−175.8	−177.3	−178.5	−176.4
HFCO	−292.9	−292.1	−289.1	−304.0	−298.6	−300.7	−307.2
HOF	−235.1	−228.3	−230.1	−236.7	−231.3	−231.1	−235.4
LiF	−193.4	−190.4	−195.5	−192.6	−194.6	−194.8	−195.5
LiH	−128.1	−130.2	−131.4	−126.4	−128.0	−131.2	−127.2
N_2	−189.1	−181.2	−186.9	−201.6	−198.4	−202.2	−205.2
N_2O	−332.8	−326.3	−328.4	−336.6	−331.8	−333.9	−339.1
NH_3	−291.9	−288.2	−291.0	−289.3	−287.8	−291.4	−290.3
OCS	−578.3	−570.7	−574.3	−585.6	−575.8	−579.8	−584.1
O_3	492.9	413.4	570.8	336.6	176.9	238.5	121.5
OF_2	−243.0	−228.5	−230.3	−250.2	−234.8	−234.1	−247.1
PN	−259.9	−249.4	−271.6	−295.5	−287.7	−292.5	−308.2
SO_2	−276.7	−275.4	−268.5	−296.1	−290.9	−295.8	−314.3

Table S8: Magnetizabilities computed with LH functionals using MS formulation of τ.

Molecule	LH07s-SVWN	LH07t-SVWN	LH12ct-SsifPW92	LH12ct-SsirPW92	CCSD(T)
AlF	−398.9	−397.2	−400.9	−399.6	−394.5
C_2H_4	−336.8	−339.5	−343.0	−342.1	−345.6
C_3H_4	−468.4	−470.1	−475.1	−473.5	−478.9
CH_2O	−112.7	−121.2	−122.2	−122.8	−127.4
CH_3F	−316.4	−316.2	−319.1	−318.1	−315.7
CH_4	−321.6	−319.3	−323.0	−321.3	−316.9
CO	−205.9	−206.9	−207.1	−207.1	−209.5
FCCH	−442.9	−442.4	−445.6	−444.6	−441.6
FCN	−369.4	−369.7	−372.2	−371.5	−370.0
H_2C_2O	−428.1	−427.2	−431.9	−430.2	−423.9
H_2O	−237.6	−237.3	−237.5	−237.3	−235.1
H_2S	−459.4	−459.0	−463.6	−461.7	−455.1
H_4C_2O	−533.5	−533.1	−539.5	−537.3	−535.2
HCN	−268.0	−270.1	−271.9	−271.4	−271.8
HCP	−484.1	−487.7	−491.2	−490.4	−492.8
HF	−178.8	−178.4	−178.1	−178.1	−176.4
HFCO	−301.0	−302.4	−304.2	−303.9	−307.2
HOF	−232.9	−234.5	−237.1	−236.4	−235.4
LiF	−197.5	−197.3	−197.6	−197.5	−195.5
LiH	−134.3	−126.4	−127.4	−126.8	−127.2
N_2	−200.3	−202.5	−203.0	−202.9	−205.2
N_2O	−336.8	−338.4	−341.0	−340.2	−339.1
NH_3	−293.3	−292.8	−294.3	−293.6	−290.3
OCS	−583.1	−584.6	−589.4	−588.0	−584.1
O_3	256.0	207.3	227.4	220.2	121.5
OF_2	−235.8	−240.4	−246.8	−245.2	−247.1
PN	−288.2	−296.8	−299.2	−298.9	−308.2
SO_2	−294.0	−298.5	−300.4	−300.0	−314.3

Table S9: Magnetizabilities computed with LH functionals using MS formulation of τ.

Molecule	LH14t-calPBE	LH20t	LH20t nonCal	LHJ14	mPSTS-a1	mPSTS-noa2	CCSD(T)
AlF	−397.3	−399.7	−399.5	−396.6	−392.2	−392.1	−394.5
C_2H_4	−340.9	−343.6	−341.5	−335.9	−333.4	−333.9	−345.6
C_3H_4	−470.2	−472.1	−472.1	−464.8	−461.1	−461.8	−478.9
CH_2O	−122.4	−126.1	−123.7	−108.1	−122.9	−124.0	−127.4
CH_3F	−315.7	−316.6	−317.5	−312.7	−311.1	−311.6	−315.7
CH_4	−317.8	−316.6	−318.9	−319.6	−310.7	−310.3	−316.9
CO	−206.9	−206.7	−205.0	−205.2	−203.5	−203.2	−209.5
FCCH	−442.5	−443.1	−442.6	−439.0	−437.9	−438.4	−441.6
FCN	−369.8	−370.4	−369.5	−366.3	−365.4	−365.8	−370.0
H_2C_2O	−425.5	−424.5	−426.6	−421.3	−419.2	−419.6	−423.9
H_2O	−236.9	−236.7	−236.4	−238.0	−234.2	−233.8	−235.1
H_2S	−457.5	−457.5	−459.8	−455.5	−449.6	−449.2	−455.1
H_4C_2O	−533.1	−536.6	−536.8	−528.6	−524.3	−525.2	−535.2
HCN	−270.7	−271.5	−269.4	−267.6	−264.6	−264.8	−271.8
HCP	−489.3	−491.1	−487.8	−482.4	−481.2	−481.6	−492.8
HF	−178.3	−177.9	−177.5	−179.6	−177.0	−176.6	−176.4
HFCO	−303.1	−304.4	−302.8	−299.4	−298.2	−298.4	−307.2
HOF	−234.3	−236.1	−235.7	−229.8	−231.3	−232.0	−235.4
LiF	−196.7	−196.4	−196.6	−196.8	−195.6	−195.5	−195.5
LiH	−125.5	−124.6	−124.7	−133.4	−127.0	−127.1	−127.2
N_2	−202.6	−202.1	−200.3	−200.4	−197.9	−197.6	−205.2
N_2O	−337.8	−337.8	−337.9	−333.1	−332.2	−332.5	−339.1
NH_3	−292.0	−291.8	−292.2	−292.6	−287.1	−286.7	−290.3
OCS	−584.5	−586.4	−586.0	−577.9	−575.9	−576.7	−584.1
O_3	207.2	223.1	229.9	244.3	175.5	183.8	121.5
OF_2	−240.5	−245.7	−245.0	−228.4	−234.8	−236.9	−247.1
PN	−297.4	−297.7	−294.4	−286.8	−286.8	−286.9	−308.2
SO_2	−299.3	−299.0	−297.0	−294.1	−289.9	−289.5	−314.3

Table S10: Magnetizabilities computed with mGGA functionals using MS formulation of τ.

Molecule	PW6B95	M06-L	MN15-L	τ-HCTH	TPSS	VSXC	B97M-V	CCSD(T)
AlF	−393.8	−382.7	−410.2	−394.1	−393.9	−399.1	−396.1	−394.5
C_2H_4	−332.8	−327.1	−343.2	−331.4	−332.1	−333.4	−334.8	−345.6
C_3H_4	−460.4	−461.5	−470.4	−458.0	−458.5	−461.1	−460.6	−478.9
CH_2O	−108.8	−123.6	−142.4	−115.4	−120.0	−116.1	−132.8	−127.4
CH_3F	−312.5	−311.1	−314.1	−308.9	−309.5	−310.3	−309.4	−315.7
CH_4	−316.5	−309.4	−318.8	−313.3	−311.5	−319.0	−313.2	−316.9
CO	−201.2	−195.3	−215.3	−203.9	−204.7	−207.5	−208.5	−209.5
FCCH	−437.3	−434.0	−450.2	−435.9	−436.7	−439.7	−440.5	−441.6
FCN	−364.0	−359.9	−376.7	−363.3	−364.8	−367.0	−368.3	−370.0
H_2C_2O	−418.4	−418.8	−438.1	−418.9	−417.9	−425.8	−425.9	−423.9
H_2O	−234.9	−230.2	−235.8	−233.8	−235.7	−235.7	−233.4	−235.1
H_2S	−453.9	−447.0	−459.0	−449.8	−450.6	−456.4	−452.3	−455.1
H_4C_2O	−528.2	−521.1	−530.1	−519.7	−521.3	−521.0	−519.7	−535.2
HCN	−264.5	−252.6	−277.0	−263.3	−264.4	−266.8	−268.9	−271.8
HCP	−479.8	−462.9	−508.5	−479.3	−480.5	−484.5	−485.9	−492.8
HF	−176.8	−173.8	−176.9	−176.3	−178.4	−177.6	−176.1	−176.4
HFCO	−295.9	−292.4	−309.7	−296.4	−297.8	−299.5	−302.5	−307.2
HOF	−230.9	−230.0	−239.5	−225.6	−229.3	−227.2	−231.5	−235.4
LiF	−193.7	−191.8	−197.3	−192.6	−195.3	−196.2	−195.6	−195.5
LiH	−131.1	−127.7	−137.9	−134.3	−129.5	−135.9	−132.4	−127.2
N_2	−196.5	−186.4	−208.7	−197.1	−198.7	−201.6	−203.2	−205.2
N_2O	−331.0	−329.0	−340.0	−329.5	−330.8	−333.1	−333.0	−339.1
NH_3	−289.7	−283.0	−291.9	−287.5	−288.4	−290.5	−287.0	−290.3
OCS	−575.5	−569.4	−590.7	−572.0	−573.6	−577.2	−577.6	−584.1
O_3	288.3	156.2	63.6	177.0	151.1	155.3	99.3	121.5
OF_2	−235.2	−234.3	−255.0	−221.6	−228.9	−224.5	−240.0	−247.1
PN	−279.1	−267.0	−330.5	−285.9	−287.3	−292.0	−301.9	−308.2
SO_2	−285.3	−284.6	−318.0	−287.5	−291.3	−296.8	−301.1	−314.3

Table S11: Magnetizabilities computed with RSH functionals using MS formulation of τ.

Molecule	ωB97M-V	ωB97X-V	ωLH22t	CCSD(T)
AlF	−400.1	−396.4	−398.7	−394.5
C_2H_4	−340.5	−338.9	−341.7	−345.6
C_3H_4	−469.9	−469.9	−472.7	−478.9
CH_2O	−115.8	−118.6	−119.6	−127.4
CH_3F	−315.0	−314.9	−317.3	−315.7
CH_4	−321.0	−317.7	−320.5	−316.9
CO	−210.5	−207.4	−207.0	−209.5
FCCH	−444.6	−442.4	−443.1	−441.6
FCN	−371.8	−370.1	−370.3	−370.0
H_2C_2O	−427.2	−425.3	−425.8	−423.9
H_2O	−237.4	−236.0	−237.6	−235.1
H_2S	−458.2	−454.2	−459.4	−455.1
H_4C_2O	−533.9	−532.8	−537.0	−535.2
HCN	−274.4	−270.7	−271.3	−271.8
HCP	−491.0	−486.5	−487.8	−492.8
HF	−178.7	−178.1	−178.6	−176.4
HFCO	−304.0	−303.1	−303.2	−307.2
HOF	−235.3	−234.7	−235.9	−235.4
LiF	−196.3	−196.4	−197.2	−195.5
LiH	−133.9	−127.4	−127.3	−127.2
N_2	−207.1	−202.9	−202.8	−205.2
N_2O	−337.5	−336.8	−338.0	−339.1
NH_3	−292.8	−290.5	−293.3	−290.3
OCS	−586.3	−584.7	−586.3	−584.1
O_3	225.3	251.2	240.9	121.5
OF_2	−245.4	−243.8	−245.0	−247.1
PN	−300.7	−296.0	−296.1	−308.2
SO_2	−303.3	−299.5	−299.1	−314.3

Table S12: Magnetizabilities computed with scLH functionals using MS formulation of τ.

Molecule	scLH22ta	scLH22t	scLH21ct-SVWN-m	scLH23t-mBR	scLH23t-mBR-P	CCSD(T)
AlF	−404.1	−399.8	−401.1	−399.7	−399.8	−394.5
C_2H_4	−340.5	−342.8	−339.3	−343.5	−343.6	−345.6
C_3H_4	−469.9	−471.0	−469.0	−472.1	−472.0	−478.9
CH_2O	−126.7	−126.6	−129.9	−126.0	−126.4	−127.4
CH_3F	−314.9	−316.2	−313.7	−316.5	−316.5	−315.7
CH_4	−317.0	−316.7	−315.8	−316.7	−316.7	−316.9
CO	−209.2	−207.1	−211.2	−206.7	−206.8	−209.5
FCCH	−440.8	−443.0	−442.9	−443.1	−443.1	−441.6
FCN	−369.7	−370.6	−372.0	−370.4	−370.5	−370.0
H_2C_2O	−424.4	−424.8	−428.0	−424.5	−424.6	−423.9
H_2O	−238.8	−236.7	−238.3	−236.7	−236.7	−235.1
H_2S	−458.7	−457.4	−456.5	−457.7	−457.8	−455.1
H_4C_2O	−534.2	−535.8	−529.1	−536.4	−536.5	−535.2
HCN	−270.1	−271.7	−272.4	−271.5	−271.6	−271.8
HCP	−486.7	−491.5	−490.5	−493.6	−493.0	−492.8
HF	−179.8	−177.9	−179.5	−177.9	−177.9	−176.4
HFCO	−306.5	−304.7	−307.1	−304.4	−304.5	−307.2
HOF	−234.4	−235.9	−234.6	−236.0	−236.0	−235.4
LiF	−199.1	−196.5	−200.3	−196.4	−196.4	−195.5
LiH	−128.7	−134.0	−134.1	−132.9	−138.2	−127.2
N_2	−202.1	−202.4	−205.5	−202.1	−202.2	−205.2
N_2O	−337.4	−338.1	−340.0	−338.0	−338.0	−339.1
NH_3	−293.1	−291.7	−292.4	−291.8	−291.8	−290.3
OCS	−584.6	−586.7	−586.1	−586.4	−586.5	−584.1
O_3	90.8	109.5	90.8	121.4	136.5	121.5
OF_2	−240.4	−245.7	−239.4	−245.7	−245.7	−247.1
PN	−303.6	−303.7	−307.9	−307.9	−303.7	−308.2
SO_2	−310.6	−302.3	−311.7	−299.4	−300.1	−314.3

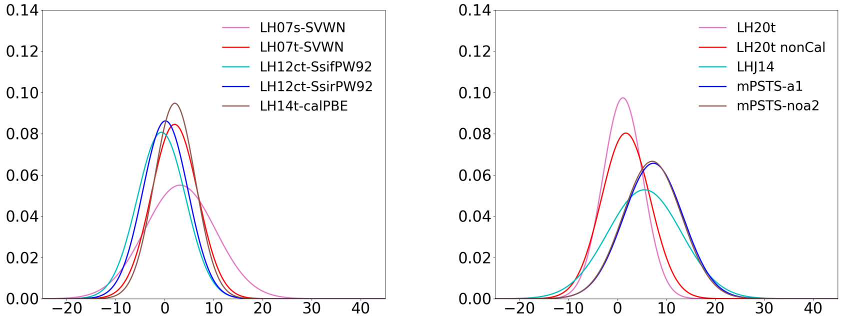

(a) LH functionals

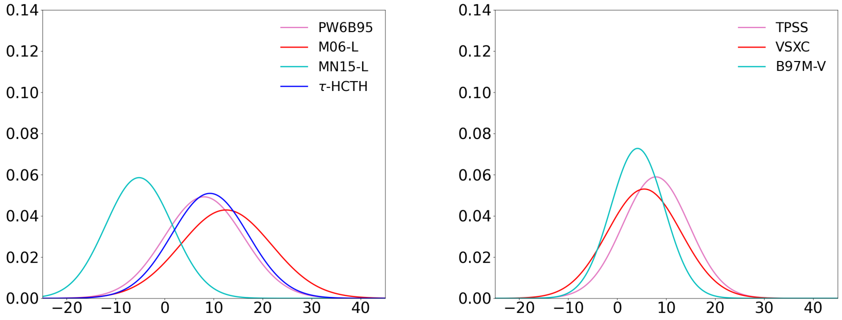

(b) mGGA functionals

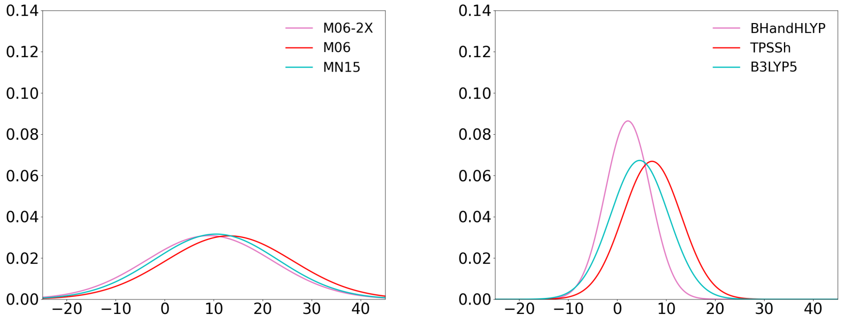

(c) GH functionals

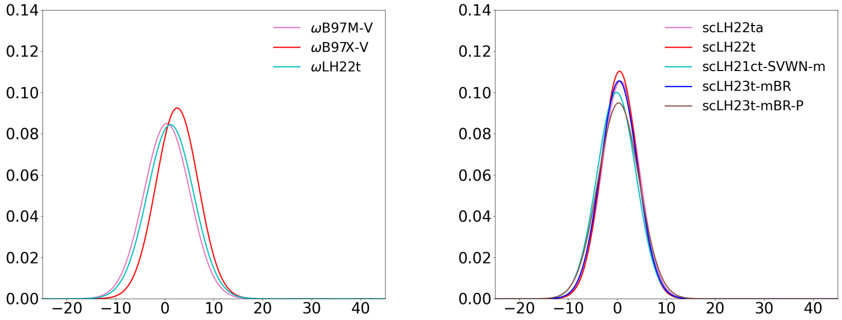

(d) RSH and scLH functionals

Figure S2: Normal distributions of the magnetizability data calculated with the Maximoff–Scuseria formulation of τ.

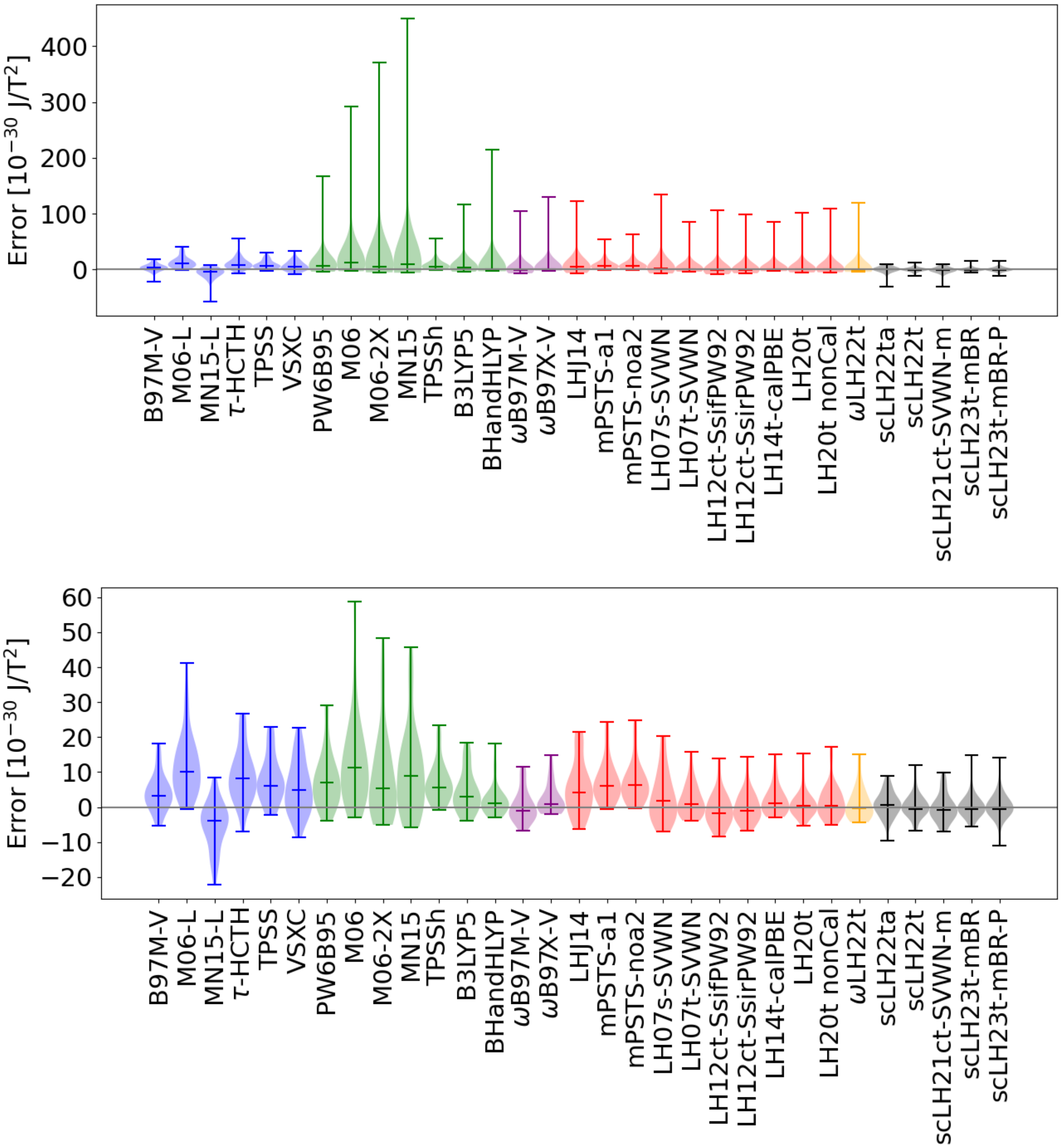

Figure S3: Violin plots of the error distribution with (top) and without (bottom) ozone using the Maximoff–Scuseria formulation of τ for meta-GGA functionals.

S1 Implementation of magnetic field derivatives for scLHs and RSLHs

S1.1 Calculation of Shielding Tensors with Hybrid Functionals

The calculation of magnetizabilities and shielding constants both require an expression for the magnetically perturbed density

$$\frac{\partial D^{\sigma}_{\mu\nu}}{\partial \mathbf{B}} = \left(D^{\sigma}_{\mu\nu}\right)^{\mathbf{B}} = \left(\sum_i c_{\mu i,\sigma} c_{\nu i,\sigma}\right)^{\mathbf{B}}, \quad (1)$$

which is constructed from the perturbative orbital rotation coefficients (u_{ia}), which are solved from the coupled-perturbed equations (CPEs)

$$u^{\mathbf{B}}_{ai,\sigma}\left(\varepsilon_a - \varepsilon_i\right) = F^{\mathbf{B}}_{ai,\sigma} - \varepsilon_i S^{\mathbf{B}}_{ai,\sigma}. \quad (2)$$

The CPEs require an expression for the perturbed Fock matrix, which depends implicitly on the perturbed XC potential $F^{\mathbf{B}}_{ai,\sigma}\left[\ldots, \left(V^{\mathrm{XC}}_{ai,\sigma}\right)^{\mathbf{B}}\right]$, and hence on the chosen XC functional. The perturbed potential is defined as

$$\left(V^{\mathrm{XC}}_{\mu\nu,\sigma}\right)^{\mathbf{B}} = \left(\frac{\partial E_{\mathrm{XC}}}{\partial D^{\sigma}_{\mu\nu}}\right)^{\mathbf{B}}. \quad (3)$$

For any global or local hybrid functional, this can be separated into (i) a semi-local contribution

$$\left(V^{\mathrm{LH,sl.}}_{\mu\nu,\varsigma}\right)^{\mathbf{B}} = \sum_{Q \varepsilon \mathbb{Q}} \int \frac{\partial E_{XC}}{\partial Q} \left(\frac{\partial Q}{\partial D^{\varsigma}_{\mu\nu}}\right)^{\mathbf{B}} \mathrm{d}\mathbf{r}, \quad (4)$$

and (ii) an exact-exchange contribution

$$\left(V^{\mathrm{LH,ex.}}_{\mu\nu,\varsigma}\right)^{\mathbf{B}} = \int \sum_{\sigma} \frac{\partial E_{\mathrm{XC}}}{\partial e^{\mathrm{ex.}}_{\mathrm{X},\sigma}} \left(\frac{\partial e^{\mathrm{ex.}}_{\mathrm{X},\sigma}}{\partial D^{\varsigma}_{\mu\nu}}\right)^{\mathbf{B}} \mathrm{d}\mathbf{r}. \quad (5)$$

$\mathbb{Q} = \mathbb{Q}\left[\rho_\sigma, \nabla\rho_\sigma, \tau_\sigma, \Delta\rho_\sigma, \nabla\nabla^{\mathrm{T}}\rho_\sigma\right]$ in eq. (4) contains the semi-local quantities present in the underlying semi-local exchange functional, as well as in the calibration function (CF) and local mixing function (LMF) in the case of local hybrid (LH), strong-correlation corrected LH (scLH) functionals, and range-separated LH (RSLH) functionals.

S1.2 Extension of the Derivatives to LHs, scLHs, and RSLHs

The derivatives for LHs have been outlined in detail in refs. S1–S3. We reproduce them here for clarity. The semi-local contribution reads

$$\frac{\partial e^{\mathrm{LH}}_{\mathrm{XC}}}{\partial Q} = (1-g)\frac{\partial e^{\mathrm{sl.},*}_{\mathrm{X},\sigma}}{\partial Q} - \left(e^{\mathrm{sl.},*}_{\mathrm{X},\sigma} - e^{\mathrm{ex.}}_{\mathrm{X},\sigma}\right)\frac{\partial g}{\partial Q} + \frac{\partial e_{\mathrm{C}}}{\partial Q}, \quad (6)$$

where $e^{\mathrm{sl.},*}_{\mathrm{X},\sigma}$ denotes a semi-local exchange-energy density augmented by an also semi-local calibration function, i.e. $e^{\mathrm{sl.},*}_{\mathrm{X},\sigma} = e^{\mathrm{sl.}}_{\mathrm{X},\sigma} + G_\sigma$.

The exact-exchange contribution reads

$$\frac{\partial e^{\mathrm{LH}}_{\mathrm{XC}}}{\partial e^{\mathrm{ex.}}_{\mathrm{X},\sigma}} = g(\mathbf{r}) \quad (7)$$

which is simply the LMF.

Extending these derivatives to a general scLH, we find

$$\frac{\partial e^{\mathrm{scLH}}_{\mathrm{XC}}}{\partial Q} = 2\Bigg[q_{\mathrm{AC}}\left((1-g)\frac{\partial e^{\mathrm{sl.},*}_{\mathrm{X},\sigma}}{\partial Q} + \left(e^{\mathrm{ex.}}_{\mathrm{X},\sigma} - e^{\mathrm{sl.},*}_{\mathrm{X},\sigma}\right)\frac{\partial g}{\partial Q} + \frac{\partial e_{\mathrm{C}}}{\partial Q}\right) + \left((1-g)\left(e^{\mathrm{sl.},*}_{\mathrm{X},\sigma} - e^{\mathrm{ex.}}_{\mathrm{X},\sigma}\right) + e_{\mathrm{C}}\right)\frac{\partial q_{\mathrm{AC}}}{\partial Q}\Bigg], \quad (8)$$

and

$$\frac{\partial e^{\mathrm{scLH}}_{\mathrm{XC}}}{\partial e^{\mathrm{ex.}}_{\mathrm{X},\varsigma}} = 2\left[\sum_{\sigma}(1-g)\left(e^{\mathrm{sl.},*}_{\mathrm{X},\sigma} - e^{\mathrm{ex.}}_{\mathrm{X},\sigma}\right) + e_{\mathrm{C}}\right]\frac{\partial q_{\mathrm{AC}}}{\partial e^{\mathrm{ex.}}_{\mathrm{X},\varsigma}} - 2\,(1-g)\cdot q_{\mathrm{AC}}. \quad (9)$$

Setting q_{AC} to the constant value $q_{AC} = 0.5$ reduces eqs. (8) and (9) to eqs. (6) and (7), respectively. Hence, the scLH extension requires a different weighting of the perturbed semi-local and exact-exchange integrals.

RSLHs use a separation of the semi-local and exact-exchange contributions into short-range and long-range contributions

$$e_{\mathrm{X}} = e_{\mathrm{X}}^{\mathrm{SR}} + e_{\mathrm{X}}^{\mathrm{LR}}. \tag{10}$$

The semi-local LR-contribution is screened, hence

$$\frac{\partial e_{\mathrm{XC}}^{\mathrm{RSLH}}}{\partial Q} = (1-g)\frac{\partial e_{\mathrm{X},\sigma}^{\mathrm{sl.,SR,*}}}{\partial Q} - \left(e_{\mathrm{X},\sigma}^{\mathrm{sl.,SR,*}} - e_{\mathrm{X,SR},\sigma}^{\mathrm{ex.}}\right)\frac{\partial g}{\partial Q} + \frac{\partial e_{\mathrm{C}}}{\partial Q}. \tag{11}$$

Such potential contributions have been implemented previously as described in ref. S4, and have to be contracted with the same semi-local quantities Q as the standard LH functionals. The potential contribution of the range-separated exact exchange is given by

$$\left(V_{\mu\nu,\varsigma}^{\mathrm{RSLH,ex.}}\right)^{\mathbf{B}} = \sum_{\sigma}\int\left[g(\mathbf{r})\cdot\left(\frac{\partial e_{\mathrm{X},\sigma}^{\mathrm{ex.,SR},\omega}}{\partial D_{\mu\nu}^{\varsigma}}\right)^{\mathbf{B}} + \left(\frac{\partial e_{\mathrm{X},\sigma}^{\mathrm{ex.,LR},\omega}}{\partial D_{\mu\nu}^{\varsigma}}\right)^{\mathbf{B}}\right]\mathrm{d}\mathbf{r}, \tag{12}$$

which in contrast to the LH or scLH expressions requires the construction of the second derivatives of the SR and LR exact-exchange expression with respect to the density matrix and magnetic field. For example, the SR exact-exchange part is given by

$$e_{\mathrm{X}}^{\mathrm{ex.,SR},\omega} = -\frac{1}{2}\sum_{\mu\nu\kappa\lambda} D_{\mu\nu}^{\sigma}D_{\kappa\lambda}^{\sigma}\omega_{\mu}^{*}(\mathbf{r})\omega_{\lambda}(\mathbf{r})A_{\kappa\nu}^{\mathrm{SR},\omega}(\mathbf{r}), \tag{13}$$

where

$$A_{\kappa\nu}^{\mathrm{SR},\mu}(\mathbf{r}) = \int \mathrm{erf}\left(\mu|\mathbf{r}-\mathbf{r}'|\right)\frac{\omega_{\kappa}^{*}(\mathbf{r}')\omega_{\nu}(\mathbf{r}')}{|\mathbf{r}-\mathbf{r}'|}\mathrm{d}\mathbf{r}. \tag{14}$$

The LMF weighs the SR contribution, and hence the perturbed SR exact-exchange contribution requires the construction of the perturbed A matrix. But as the magnetic field only acts on the GIAOs, we find

$$\left(A_{\kappa\nu}^{\mathrm{SR},\mu}\right)^{\mathbf{B}_k} = \frac{i}{2c}\left(\mathbf{R}_{\mu\nu}\times\left(\mathbf{A}_{\mu\nu}^{\mathrm{SR},\mu,l_{k+1}} + \mathbf{R}_K A_{\mu\nu}^{\mathrm{SR},\mu}\right)\right)_k, \tag{15}$$

which can be constructed from standard RS exact-exchange integrals with an enlarged l-quantum number for the first A-matrix contribution. In eq. (15), $k = x, y, z$, $\mathbf{R}_{\mu\nu}$ is the distance vector between the corresponding AOs, and $\mathbf{R}_K$ the position of the nucleus of choice.

References

(S1) Schattenberg, C. J.; Reiter, K.; Weigend, F.; Kaupp, M. An Efficient Coupled-Perturbed Kohn–Sham Implementation of NMR Chemical Shift Computations with Local Hybrid Functionals and Gauge-Including Atomic Orbitals. *J. Chem. Theory Comput.* **2020**, *16*, 931–943.

(S2) Schattenberg, C. J.; Kaupp, M. Effect of the Current Dependence of Tau-Dependent Exchange-Correlation Functionals on Nuclear Shielding Calculations. *J. Chem. Theory Comput.* **2021**, *17*, 1469–1479.

(S3) Schattenberg, C. J.; Kaupp, M. Implementation and Validation of Local Hybrid Functionals with Calibrated Exchange-Energy Densities for Nuclear Shielding Constants. *J. Phys. Chem. A* **2021**, *125*, 2697–2707.

(S4) Fürst, S.; Haasler, M.; Grotjahn, R.; Kaupp, M. Full Implementation, Optimization, and Evaluation of a Range-Separated Local Hybrid Functional with Wide Accuracy for Ground and Excited States. *J. Chem. Theory Comput.* **2023**, *19*, 488–502.
